# Subclinical perfusion deficits in patients with Type 2 diabetes detectable with Cardiovascular Magnetic Resonance Imaging

**DOI:** 10.1186/1532-429X-13-S1-P129

**Published:** 2011-02-02

**Authors:** Anna Schmidt, David Lau, Matthias G Friedrich

**Affiliations:** 1 Stephenson CMR Center, University of Calgary, Calgary, AB, Canada; 2 Stephenson CMR Centre , University of Calgray, Calgary, AB, Canada; 3 Stephenson CMR Centre, University of Calgary, Calgary, AB, Canada

## Background

An estimated 200 million people worldwide have type 2 diabetes. Type 2 diabetes is recognized as an independent risk factor for adverse cardiac events, such as myocardial infarction. Cardiovascular disease is the leading cause of death amongst diabetic patients. The subclinical pathophysiology of diabetic heart disease predicts global microvascular disease, prior to the onset of overt ischemic heart disease. Non-invasive screening methods are therefore important for risk stratification of asymptomatic patients. Cardiovascular Magnetic Resonance Imaging (CMR) is a valuable tool for the assessment of subclinical microvascular function in this high-risk population.

## Objectives

To assess the degree of cardiac microvascular dysfunction in individuals type 2 diabetes (T2D) without coronary disease or hypertension, compared to healthy, non-diabetic controls.

## Methods

This cross sectional pilot data included 2 groups of subjects: Patients diagnosed with type 2 diabetes (Hb_A1c_ 7.5-9.9%; mean age 59.3±7.17; n=6), and healthy, non-diabetic age-matched controls (mean age 51.9±10.3; n=10). Medical history and ECG were reviewed to rule out ischemic heart disease. Qualified patients underwent a CMR-Adenosine stress perfusion,

## Results

**Figure 1:**
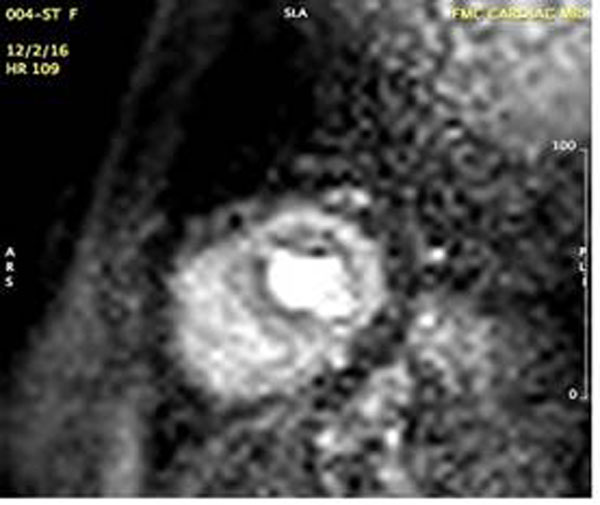
Stress perfusion image from patient diagnosed with a type 2 diabetes in 2009. Note circumferential subendocardial perfusion delay.

Subendocardial perfusion delays were observed in 4 out of 6 diabetic patients, and 0 of 10 healthy controls (p < 0.05). Importantly, the T2D patients exhibited primarily diffuse or circumferential perfusion deficits (Figure 1). Controls were normotensive (125.3/79.7 mmHg ± 6.1/2.8 mmHg), and patients had controlled blood pressure (mean 132.2/79.8 mmHg ± 8.5/5.2 mmHg), primarily through ACE inhibitors. Patients and controls had normal systolic function (LV EF 57.7 ± 3.01 and 57.8 ± 5.4, respectively).

## Conclusion

The observed perfusion abnormality supports previous nuclear imaging findings and pathophysiological research of diabetic heart disease and may indicate more diffuse patterns of microvascular disease. Further studies are required to assess the pathophysiologic context and prognostic impact of these findings.

